# Needle and syringe sharing among Iranian drug injectors

**DOI:** 10.1186/1477-7517-6-21

**Published:** 2009-07-30

**Authors:** Hassan Rafiey, Hooman Narenjiha, Peymaneh Shirinbayan, Roya Noori, Morteza Javadipour, Mohsen Roshanpajouh, Mercedeh Samiei, Shervin Assari

**Affiliations:** 1 Iranian Research Center for Substance Abuse and Dependence (IRCSAD), University of Social Welfare and Rehabilitation Science, Tehran, Iran; 2 Drug Control Head Quarters (DCHQ), Tehran, Iran; 3 Medicine and Health Promotion Institute, Tehran, Iran

## Abstract

**Objective:**

The role of needle and syringe sharing behavior of injection drug users (IDUs) in spreading of blood-borne infections – specially HIV/AIDS – is well known. However, very little is known in this regard from Iran. The aim of our study was to determine the prevalence and associates of needle and syringe sharing among Iranian IDUs.

**Methods:**

In a secondary analysis of a sample of drug dependents who were sampled from medical centers, prisons and streets of the capitals of 29 provinces in the Iran in 2007, 2091 male IDUs entered. Socio-demographic data, drug use data and high risk behaviors entered to a logistic regression to determine independent predictors of lifetime needle and syringe sharing.

**Results:**

749(35.8%) reported lifetime experience of needle and syringe sharing. The likelihood of lifetime needle and syringe sharing was increased by female gender, being jobless, having illegal income, drug use by family members, pleasure/enjoyment as causes of first injection, first injection in roofless and roofed public places, usual injection at groin, usual injection at scrotum, lifetime experience of nonfatal overdose, and history of arrest in past year and was decreased by being alone at most injections.

**Conclusion:**

However this data has been extracted from cross-sectional design and we can not conclude causation, some of the introduced variables with association with needle and syringe sharing may be used in HIV prevention programs which target reducing syringe sharing among IDUs.

## Introduction

Human Immunodeficiency Virus (HIV)/Acquired Immunodeficiency disorder syndrome (AIDS) has shown a rapid increasing trend [1]. This problem is closely associated to injecting drug users (IDUs) in Iran, accounting for 67% of HIV positive cases and 85% of AIDS cases [2].

HIV studies in Iran have underscored the sharing injecting equipments as the main routes of transmission [3]. In one study, lifetime and last time needle and syringe sharing was reported by 50% and 25% of IDUs, respectively [4]. In another study, in a drug treatment sample, more than two-thirds of the IDUs had shared syringes [5].

Identifying factors associated with needle and syringe sharing among IDUs is particularly important for HIV prevention [6]. While very little is known about associated factors of needle and syringe sharing among Iranian IDUs [7-9], we here aimed to determine the prevalence and associates of needle and syringe sharing among a sample of IDUs in Iran.

## Methods

### Design and setting

This is a secondary analysis of a cross-sectional survey on 7,743 individuals as a rapid situation assessment (RSA) performed by the Darius institute. Grant was awarded by the Iranian Research Center for Substance Use and Dependence (DARIUS Institute) affiliated to the University of Social Welfare and Rehabilitation Sciences. The study was approved by the ethical review committee of the university and informed consent was obtained from all the participants after they had been verbally reassured that the information would be kept confidential, especially from correctional system. This study was conducted under the financial aid of the Drugs Control Headquarters (DCHQ). Some other manuscripts have been extracted from this database.

### Samples and sampling

The participants were substance dependent persons according to DSM-IV and sampled from treatment centers (n = 1,217), prisons (n = 584) and streets (n = 5,860) of the capitals of 29 provinces in the Islamic Republic of Iran. The samples from treatment centers were selected at random from newcomers. Prisons sampling was also carried out randomly among those who were registered into the prison within previous 30 days. Snowball approach was used to take sample from streets. The number of samples taken from every province was proportional to the whole population of the province. The sampling started in April 2007 and lasted for 5 months. This sampling method is used as the main sampling strategy of drug use in DCHQ studies.

### Process

The interviews were carried out by university graduates (MS, BS) with drug abuse related majors/degrees who were dispatched to the provinces after being trained through workshops in Tehran (the capital of Islamic Republic of Iran). Each interview took 1 to 1 and a half hour. Data were collected using paper-based questionnaire namely Inventory for Drug Dependency-IV, which was the modified version of the one used in the previous national RSA of Iran performed by the research center [10]. The revision was done through a series of expert panel meetings, and new items and questions were added that met the desired objectives. Sixty nine items were classified in 9 different parts including: 1) socioeconomic data (at the time of data collection), 2) family data, 3) first use data, 4) lifetime drug use, 5) current drug of dependency, 6) injection data, 7) high risk behavior, 8) treatment data, and 9) social network.

### Independent data

Data included in this study included the following parts:

I) socio-demographic data: Data consisted age, age of beginning addiction, age of beginning injection, duration of injection, gender, educational level, marital status, living place, status of home, status of employment, alone living, income, legal income, illegal income, drug sell income, monthly family income, cigarette smoking, family history of cigarette smoking, family history of drug use

II) Drug related data consisted monthly money that IDUs used for dominant substance, first place of drug use, first situation of drug use, most reason for first drug use, first pesrson that who suggested drug use, dominant drug that current injectors was used(type of drug), poly drug use and history of drug problems treatment.

III) Injection related data consisted first place of injection, situation of first injection, cause of first injection, frequency of injection in the past years, usual place of injection and alone injection.

IV) Non-sexual high risk behaviors consisted of history of arrest, and history of imprisonment.

To make the final costs internationally comparable, the costs which were registered in Iranian Rials were converted to purchase power parity or international Dollar (PPP$). The conversion rate for PPP$ was based on a recently published Iranian study, which had reached at an estimation of PPP$ equal to 2727 Rials according to the information from the Central Bank of Iran and the World Bank database [11].

### Outcome

Lifetime needle and syringe sharing was defined as borrowing or lending syringe, needle or other injection equipments at least once in their life [12-14]. The most important cause for needle and syringe sharing was also included, with a multiple choice question. Answers included "no access to sterile syringes", "to get more pleasure", "quick injection", "being sure at the shared syring", "financial limitations", "not aware of possible risk", "easy injection" and "peer pressure" [15-17].

### Statistical analysis

The data obtained in the SPSS for Windows 13 statistical package. In order to present quantitative data, median (percentile 25% = Q1 and percentile 75% = Q3), mean and standard deviation was used. In order to compare the qualitative variables between those with and without "needle and syringe sharing", chi-square test was used. The comparison of age between two groups was done with t-test and expenditures of drug use between two groups with Mann-Whitney. Multivariate stepwise logistic regression was used to determine the predictors of lifetime syring sharing. P value < 0.05 was considered significant.

## Results

Mean age at study, age at first drug use, age at first injection, and duration of injection of the participants were 31.3 ± 8.3, 18.6 ± 5.4, 25.9 ± 6.7 and 7.4 ± 6.3, respectively. Most participants were Muslim, lived in urban area, single, with a lower diploma educational level.

### Needle and syringe sharing

From all 2091 IDUs, 749(35.8%) reported lifetime experience of needle and syringe sharing. Most frequent causes for needle and syringe sharing included "no access to sterile syringes" (n = 437; 20.9%), "to get more pleasure" (n = 274; 13.1%), "quick injection" (n = 164; 7.8%), "being sure at the shared syring" (n = 128; 6.1%), "financial limitations" (n = 128; 6.1%), "not aware of possible risk" (n = 99; 4.7%), "easy injection" (n = 94; 4.5%) and "peer pressure" (n = 61; 2.9%).

### Associates of Needle and syringe sharing

#### Socio-demographic data

IDUs with lifetime syring sharing had a higher mean age (32.3 ± 8.9 vs. 31.4 ± 8.1; p = 0.02), lower age of first drug use (17.9 ± 5 vs. 18.6 ± 5.4; p = 0.005), higher duration of injection (6.5 ± 6.3 vs. 5.4 ± 5.5; p < 0.001). Age at first injection was not linked to lifetime syring sharing (25.8 ± 6.8 vs. 26.1 ± 6.7; p = 0.28). IDUs with lifetime syring sharing had lower monthly family income (733 ppp$, Q1 = 330 ppp$, Q3 = 1283 ppp$ vs. 807 ppp$, Q1 = 476 ppp$, Q3 = 1466 ppp$; p < 0.001). Overall monthly paiment on drugs were not linked to lifetime syring sharing (586 ppp$, Q1 = 330 ppp$, Q3 = 1063 ppp$ vs. 550 ppp$, Q1 = 366 ppp$, Q3 = 1100 ppp$; p = 0.44). Bivariate analysis showed that needle and syringe sharing was significantly higher in females, those who lived in rural area, those who were illiterate, those who were separate/divorce/widow, homeless, those who lived alone, those jobless, those with illegal income, those with drug related income and those with drug use family members (Table 1).

**Table 1:** The comparison of syringe sharing between socio-demographic variables

		Syringe sharing		
			
		Count	Percent	P value
**Sex**	Male	713	35.4%	0.029
		
	Female	35	47.9%	

**Religious type**	Muslim	737	36.0%	0.164
		
	other	2	16.7%	

**Living place**	Urban	640	35.0%	0.004
		
	Rural	62	47.7%	

**Education level**	Illiterate or were barely able to read and write	94	43.7%	0.002
		
	Under diploma	607	36.0%	
		
	Upper diploma	35	25.0%	

**Marital status**	single	421	38.0%	<0.001
		
	Married	166	26.4%	
		
	Separate, divorce and widow	153	46.8%	

**Status of home**	Having home	575	32.4%	0.000
		
	Homeless	144	62.1	

**Alone Living**	No	567	32.8%	<0.001
		
	Yes	182	50.6%	

**Occupation**	No	353	28.5%	<0.001
		
	Yes	396	46.5%	

**Boss type**	State	13	25.0%	0.591
		
	Private	114	30.9%	
		
	khisfarrma	126	28.5%	

**Drug Income**	No	462	30.2%	<0.001
		
	Yes	267	52.0%	

**Job Income**	No	409	43.1%	<0.001
		
	Yes	320	29.3%	

**Illegal Income**	No	412	28.5%	<0.001
		
	Yes	317	53.0%	

**Legal Non Job Income**	No	345	35.3%	0.775
		
	Yes	384	36.0%	

**Furniture sell Income**	No	683	35.5%	0.483
		
	Yes	46	38.7%	

**Lifetime smoking**	Never smoking	23	34.3%	0.636
		
	Current smoking	690	36.1%	
		
	Past smoking	36	31.9%	

**cigarette smoking by parents**	No	296	29.8%	<0.001
		
	Yes	453	41.3%	

**cigarette smoking by other members of family**	No	178	26.8%	<0.001
		
	Yes	571	40.0%	

**Substance use by parents**	No	475	31.3%	<0.001
		
	Yes	274	47.7%	

**Substance use by members of family**	No	337	29.0%	<0.001
		
	Yes	412	44.3%	

#### Substance-related and injection-related data

Needle and syringe sharing was higher in IDUs who used heroin (331,41.3% vs. 418,32.4%; p < 0.001), was lower in those who used opioium (47,23.6% vs.702,37.1%; p < 0.001) and was lower in those who used Amphetamines (12,15.6% vs. 737,36.6%; p <0.001). Poly drug users was associated with needle and syringe sharing (321, 40.5% vs. 415, 33.3%; p = 0.001). (Table 2).

**Table 2:** The comparison of syringe sharing between drug use-related variables

			Syringe sharing		P value
				
			Count	Percent	
Dominant drug usage	Opioium	Yes	47	23.6%	<0.001
			
		No	702	37.1%	
		
	Amphetamines	Yes	12	15.6%	<0.001
			
		No	737	36.6%	
		
	Heroin	Yes	331	41.3%	<0.001
			
		No	418	32.4%	
		
	Purified Heroin	Yes	202	35.9%	0.943
			
		No	547	35.8%	
		
	Norjesic	Yes	109	35.6%	0.937
			
		No	640	35.9%	

First place of drug use	Own home, home of friends, student home		338	32.1%	0.003
		
	Roofless public places		225	40.7%	
		
	Roofed public places		95	36.3%	

First situation of drug use	Specific situations(family party, friends party, mourning ceremony, gatherings with friends)		559	35.5%	0.524
		
	Without Specific situation		187	37.1%	

What was the most important event that leaded you to first use?	Specific events(work related, familial/domestic, educational)		360	38.6%	0.029
		
	Without Specific event		376	34%	

Most important reason for beginning drug use	Pleasure/enjoyment, recreation, Konjkavi		402	34%	0.033
		
	Without pleasure/enjoyment		343	38.6%	

First person who suggested you to use substance	Family or relatives		149	42.2%	0.015
		
	Friends		377	35.5%	
		
	Assistants		45	31.5%	
		
	Others		52	29.5%	
		
	Without mover		96	32.0%	

Needle and syringe sharing was lower in those who alone inject (most of times) and home as first place of injection (Table 3).

**Table 3:** The comparison of syringe sharing between injection-related variables

			**Syringe sharinge**		
				
			**Number**	**Percent**	**P value**
First place of injection	Own home, home of friends, student home		382	31.6%	0.003
		
	Roofless public places		206	41.4%	
		
	Roofed public places		88	50%	

Frequency of injection	Lower than once per day		145	31%	0.001
		
	Once and higher per day		573	38.2%	

Site of injection	Hand	No	76	34.4%	0.639
		
		Yes	673	36.0%	
		
	Foot	No	393	29.9%	<0.001
		
		Yes	356	45.8%	
		
	Groin	No	393	29.9%	<0.001
		
		Yes	160	58.4%	
		
	Testis	No	575	32.1%	<0.001
		
		Yes	174	57.6%	
		
	Neck	No	622	33.4%	<0.001
		
		Yes	127	56.2%	
		
	Other	No	725	35.6%	0.316
		
		Yes	24	42.1%	

Cause of first injection	Speed of effect	No	446	32.9%	<0.001
			
		Yes	303	41.2%	
		
	pleasure/enjoyment	No	404	30.7%	<0.001
			
		Yes	345	44.5%	
		
	Effect less of before mode of drug use	No	526	33.3%	<0.001
			
		Yes	223	43.6%	
		
	Pry	No	589	35.9%	0.864
			
		Yes	160	35.5%	
		
	Relief of use	No	573	35.2%	0.265
			
		Yes	176	38.0%	
		
	Pressure of friends	No	610	35.2%	0.208
			
		Yes	139	38.7%	
		
	Substance was not out of reach	No	668	35.0%	0.007
			
		Yes	81	45.0%	
		
	Low quality of present drugs	No	665	34.5%	<0.001
			
		Yes	84	52.2%	
		
	Lower cost of injection	No	603	32.7%	<0.001
			
		Yes	146	59.6%	
		
	Treatment of addiction	No	736	36.2%	0.031
			
		Yes	13	22.4%	

Where do you usually inject?	Own's home	No	375	39.8%	0.001
			
		Yes	374	32.6%	
		
	Park	No	575	33.3%	<0.001
			
		Yes	174	48.1%	
		
	School	No	743	35.8%	0.436
			
		Yes	6	46.2%	
		
	Street and lane	No	554	32.2%	<0.001
			
		Yes	195	52.2%	
		
	"Kharabe"	No	385	26.2%	<0.001
			
		Yes	364	58.4%	
		
	Student's house	No	740	35.7%	0.208
			
		Yes	9	50%	
		
	Soldiers' camp	No	737	35.5%	<0.001
			
		Yes	12	80%	
		
	Prison	No	663	33.5%	<0.001
			
		Yes	86	76.8%	
		
	Work place	No	661	35.5%	0.44
			
		Yes	88	38.1%	
		
	Friend's home	No	473	34.6%	0.096
			
		Yes	276	38.2%	

With whom do you usually inject?	Alone		493	33.0%	<0.001
		
	With others(friends, relatives)		256	43.0%	

#### High risk behaviors

Lifetime needle and syringe sharing was significantly higher in those IDU who reported extramarital sexual relation) 480,64.1% vs. 269,35.9%; p < 0.001), history of being arrested by police in the past year(507,67.7% vs. 242,32.3%; p < 0.001) and history of imprisonment in the past year(455,60.7% vs. 294,39.3%; p < 0.001).

#### Logistic regression

Multivariate logistic regression showed that the likelihood of lifetime needle and syringe sharing was increased by female gender(OR = 2.68, 95%CI = 1.25–5.72, p = 0.01), being jobless (OR = 1.87, 95%CI = 1.41,2.47, p = 0.001), having illegal income (OR = 1.61, 95%CI = 1.21–2.15, p < 0.001), drug use by family members (OR = 1.47, 95%CI = 1.12–1.92, p = 0.005), first drug use in roofless public place (Odds Ratio = 1.55, 95%CI = 1.15–2.09, p = 0.003), first drug use in roofed public place (Odds Ratio = 1.62, 95%CI = 1.08–2.42, p = 0.01), pleasure/enjoyment as causes of first injection (OR = 1.58, 95%CI = 1.2–2.07, p = 0.001), usual injection at groin(OR = 1.64, 95%CI = 1.11–2.42, p = 0.01), usual injection at scrotum (OR = 1.57, 95%CI = 1.06–2.31, p = 0.02), lifetime experience of nonfatal overdose (OR = 1.68, 95%CI = 1.28–2.21, p < 0.001), and history of arrest in past year (OR = 1.38, 95%CI = 1.04–1.82, p = 0.02) and was decreased by being alone at most injections (OR = 0.51, 95%CI = 0.38–0.68, p < 0.001). (Table 4).

**Table 4:** Logestic regression for having syringe sharing between socio-demographic, drug use and injection-related variables in intravenous drug users (IDUs)

			**95% Confidence Interval for odds**	
			
	**P value**	**OR**	**Lower**	**Upper**
Gender(female)			1.259	5.725

Jobless	<.001	1.870	1.412	2.478

Illegal Income	.001	1.617	1.217	2.150

Substance use of family members	.005	1.471	1.125	1.925

pleasure/enjoyment as cause of first injection	.001	1.583	1.209	2.074

First place of drug use (Roofless public places)	.003	1.558	1.157	2.097

First place of drug use (Roofed public places)	.019	1.621	1.084	2.424

Alone injection	<.001	.515	.388	.682

Groin injection	.013	1.642	1.111	2.427

Testis injection	.022	1.573	1.068	2.318

Nonfatal overdose	<.001	1.686	1.282	2.216

Arresting in past year	.022	1.385	1.049	1.829

## Discussion

In Iran, 1 of 3 IDUs report the history of lifetime needle and syringe sharing. The likelihood of lifetime needle and syringe sharing was increased by female gender, being jobless, having illegal income, drug use by family members, pleasure/enjoyment as causes of first injection, first injection in roofless and roofed public places, usual injection at groin, usual injection at scrotum, lifetime experience of nonfatal overdose, and history of arrest in past year and was decreased by being alone at most injections.

Regarding the literature on syring sharing, according to a study in Mexico, 2005, 80% of the IDUs reported that they share syringes regularly with other IDUs [18]. In another study in Canada 27.6% of the participants reported sharing needles during the past 6 months [12].

Our study showed that needle and syringe sharing was increased in female IDUs. In line with our finding, one study of gender effect on needle and syringe sharing bahavior of IDUs showed that females were more likely to share injecting equipment [19-21]. A recently qualitative study of Iranian female IDUs reported sharing syringes as a typical behavior [8]. Different risk profile of HIV among male and female IDUs is in line with these reports [22]. So, gender should be addressed as an important variable in needle exchange programs [23].

In our study, jobless IDUs and those who had illegal income had higher rate of needle and syringe sharing. Review of literature shows a link between unemployment of IDUs and needle and syringe sharing behavior [24,25]. Similarly, syringe has been reported to be linked to not having a legal income [26] or engaging in illegal jobs [27]. These may be due to the financial strains to buy stril syrings, and free syrings should be given to these IDUs.

In our study, drug injectors with drug user family members were at higherrisk for needle and syringe sharing. Needle and syringe sharing is reported to be higher in IDUs with a familial network for drug use [28]. Oe study reported that the role of family network on the needle-sharing behavior is more severe in women in comparison with men [29].

In our study, first drug use at public places was linked to more syringe sharering. According to the literature, IDUs who usually inject in public places have oppurtunity for needle and syringe sharing [30,31]. A qualitative study in Iran also confirms this association [8].

Alone injection in compare to injection with someone else, is linked to the lack of oppurtiunity of needle and syringe sharing. In one study in US, markedly lower rates of needle and syringe sharing was observed in IDUs who injecting alone [32]. Injection in the context of social and familial networks is known to be associated with higher needle and syringe sharing [28]. Those who try to keep their injecting hidden, may benefit of a reduced risk of syring sharing [32].

We found that injection in groin and linked to higher needle and syringe sharing in IDUs. However we did not find any study in this regard, studies of bodily injection sites of IDUs have reported a clear progression in sites used, from the upper extrimities, at initial injection to the use of sites such as the groin and scrotum the years after [33,34]. Unjection in sites such as the groin and scrotum were linked to a greater number of injection-related problems. One study showed a link between more severe drug injecting and share needles [25].

We found a link between needle and syringe sharing and nonfatal overdose, which are both high risk behaviors. One study in USA showed that overdosing may be associated with borrowing syringes [35] but in another study in England in1994 to 1995 self-reported overdose was not linked to syring sharing [36]. We also found arrest in the past year as a associated factor with needle and syringe sharing. Similar results have been reported by two studies in Pakistan and Australia [37,38]. Other Risk Behavior Surveys have shown a Co-occurrence of health-risk behaviors among differerent populations [39,40]. These studies have explained their findings with the gateway theory.

In Iran, evidences show that access to a needle and syringe program (NSP) will reduce the needle and needle and syringe sharing practices. The authors suggested NSPs to be intensified in settings with concentrated HIV epidemics among IDUs in Iran [13].

There are some limitations to this study. First, this study is one of a series of secondary analyses [41] and we did not have data on detail of needle and syringe sharing behaviors. Second, the results rely on participants' self-report data, because self-reports are affected by response bias. Respondents may tend to deny or underreport their syring sharing due to social disirability [41]. Third, because of the cross-sectional design of this study, it is not possible to draw a conclusion on the direction of the associations. Endly, in this study we asked lifetime syring sharing, and we did not limit it by asking sharing during past year or last injection.

## Conclusion

In designing interventions for HIV prevention in Iran, through decrease of needle and syringe sharing among IDUs, the introduced variables must be considered. Further studies in this regard are needed.

## Competing interests

The authors declare that they have no competing interests.

## Authors' contributions

SA performed the secondary analysis. MJ and MR prepared the draft of the manuscript. HN, HR helped SA in interpretation of the secondary analysis. All authors read and approved the final manuscript. HR, HN, RN, MS and PS participated in the design of the original survey.
